# A robust multiple heartbeats classification with weight-based loss based on convolutional neural network and bidirectional long short-term memory

**DOI:** 10.3389/fphys.2022.982537

**Published:** 2022-12-05

**Authors:** Mengting Yang, Weichao Liu, Henggui Zhang

**Affiliations:** ^1^ Key Laboratory of Medical Electrophysiology, Ministry of Education and Medical Electrophysiological Key Laboratory of Sichuan Province, (Collaborative Innovation Center for Prevention of Cardiovascular Diseases), Institute of Cardiovascular Research, Southwest Medical University, Luzhou, China; ^2^ School of Medical Information and Engineering, Southwest Medical University, Luzhou, China; ^3^ School of Biomedical Engineering and Instrument Science, Zhejiang University, Hangzhou, China; ^4^ Department of Physics and Astronomy, The University of Manchester, Manchester, United Kingdom

**Keywords:** electrocardiogram (ECG), deep learning, cardiac arrhythmia, convolutional neural network (CNN), bidirectional long short-term memory (bi-LSTM)

## Abstract

**Background:** Analysis of electrocardiogram (ECG) provides a straightforward and non-invasive approach for cardiologists to diagnose and classify the nature and severity of variant cardiac diseases including cardiac arrhythmia. However, the interpretation and analysis of ECG are highly working-load demanding, and the subjective may lead to false diagnoses and heartbeats classification. In recent years, many deep learning works showed an excellent role in accurate heartbeats classification. However, the imbalance of heartbeat classes is universal in most of the available ECG databases since abnormal heartbeats are always relatively rare in real life scenarios. In addition, many existing approaches achieved prominent results by removing noise and extracting features in data preprocessing, which relies heavily on powerful computers. It is a pressing need to develop efficient and automatic light weighted algorithms for accurate heartbeats classification that can be used in portable ECG sensors.

**Objective:** This study aims at developing a robust and efficient deep learning method, which can be embedded into wearable or portable ECG monitors for classifying heartbeats.

**Methods:** We proposed a novel and light weighted deep learning architecture with weight-based loss based on a convolutional neural network (CNN) and bidirectional long short-term memory (Bi-LSTM) that can automatically identify five types of ECG heartbeats according to the AAMI EC57 standard. It was also true that the raw ECG signals were simply segmented without noise removal and other feature extraction processing. Moreover, to tackle the challenge of classification bias due to imbalanced ECG datasets for different types of arrhythmias, we introduced a weight-based loss function to reduce the influence of over-weighted categories in the ECG dataset. For avoiding the influence of the division of validation dataset, k-fold method was adopted to improve the reliability of the model.

**Results:** The proposed algorithm is trained and tested on MIT-BIH Arrhythmia Database, and achieves an average of 99.33% accuracy, 93.67% sensitivity, 99.18% specificity, 89.85% positive prediction, and 91.65% F_1_ score.

## Introduction

Cardiovascular diseases (CVD) are the leading cause of death worldwide. According to the 2019 statistics from the American Heart Association, there are an estimated 23.6 million cardiac deaths in 2030 (Benjamin et al., 2019). Due to its high current incidence and predicted increasing trend in soon future, it is pressing to develop novel methods for early and accurate diagnosis/classification of cardiac diseases. Arrhythmia is a common form of cardiac diseases and sometimes life-threatening, it always leads to or occurs with others CVD. Due to the non-invasive nature of electrocardiogram (ECG), the body surface ECG serves as a convenient diagnostic method for diagnosing arrhythmia, which is almost impossible to be replaced with other methods. ECG reflects features of excitation and propagation of cardiac excitation sequences during a cardiac cycle, which is obtained by measuring the potential change of electrodes placed in different parts of the human torso, providing an effective indicator of CVD (Malmivuo, 1995). Detecting abnormal heart rhythms as early as possible not only helps save a patient’s life but also alleviates sequelae in patients, reducing the burden of healthcare. Therefore, an efficient and accurate diagnosis of ECG rhythm is important for the treatment and medical care of cardiac patients. However, it is time-consuming and laborious to identify abnormal ECG signals due to its feature of high complexity and high noise in the clinic.

Over the years, with the emergence of the Medical Internet of Things and intelligent devices, more and more wearable devices have been developed to achieve continuous and remote monitoring of ECG. In addition, improved computer hardware and more standardized data lead to the development of automatic heartbeat classification based on machine learning (ML)/artificial intelligence (AI), which has attracted increasing attention in recent years. These popular ML/AI methods include support vector machines (SVM) (Ebrahimzadeh et al., 2018; Hammad et al., 2021a; Sharma et al., 2019), deep learning (Acharya et al., 2017; Liu et al., 2021; Somani et al., 2021; Beetz et al., 2022), and so on. It is believed intelligent diagnostic system based on AI for heartbeats classification can effectively reduce the burden and possibility of subjective uncertainty of experts, which may lead to misdiagnosis. The traditional computer-aided AI arrhythmia diagnosis algorithm includes three main steps: data preprocessing, feature extraction, and ECG classification (Hammad et al., 2021a). However, the high ability of fully automatic feature extraction in deep learning makes it much easier to diagnose ECG heartbeat, which was the critical step in conventional ML. The data preprocessing involving noise removal and feature extraction are heavily relying on computer resources and limiting its use in wearable devices.

Deep learning is a series of representation layers with an automatic search process for better data representation, and these layers are learned through training processes of an artificial neural network (Chollet, 2021), which contribute to automatically extracting features and learning data representation. A recent study has shown that arrhythmia diagnosis based on deep learning can achieve higher accuracy and efficiency than expert’s manual classification (Murat et al., 2020). Over the years, many ML/AI-based algorithms with different datasets to focus on the ECG arrhythmia classification for automatic detection have been developed. A general overview of ECG arrhythmia classification using machine learning and deep learning methods is presented in (Luz et al., 2016; Kooman et al., 2020; Xie et al., 2020; Hong et al., 2021; NehaSardana et al., 2021; Merdjanovska and Rashkovska, 2022). There are many different databases available for arrhythmia research, such as PTB-XL (Wagner et al., 2020; Prabhakararao and Dandapat, 2021; Smigiel et al., 2021; Karthik et al., 2022; Palczynski et al., 2022), and MIT-BIH (Acharya et al., 2017; Goldberger et al., 2000; Sayantan et al., 2018; Nurmaini et al., 2020; Yildirim et al., 2018; Huang et al., 2019; Wang et al., 2019). In general, many well-designed methods were proposed in the past few years. Among them, Wang et al. (Wang et al., 2021) developed a novel method based on Continuous Wavelet Transform and CNN for ECG arrhythmia classification, which tested on MIT-BIH arrhythmia database and achieved an overall performance of 68.76% F_1_ score and 98.74% accuracy. Oh et al. (Oh et al., 2019) proposed a modified U-net to diagnose cardiac conditions and attained a high classification accuracy of 97.32%, and 99.3% for R peak detection using a ten-fold cross-validation strategy. Yildirim et al. (Yildirim et al., 2018) adopted the 1-D CNN model and focused on 17 arrhythmia classifications, resulting in an average accuracy of 91.33%. Prabhakararao et al. (Prabhakararao and Dandapat, 2021) designed a classifier based multiple scale-dependent deep convolutional neural networks with different receptive fields for arrhythmia classification, the model showed impressive performance (averaged 84.5% F_1_ score on PTBXL-2020 dataset and 88.3% F_1_ score on CinC-2017 dataset) and generalization ability, and then made it suitable for arrhythmia monitoring applications. Zahid et al. (Zahid et al., 2022) used MIT-BIH arrhythmia dataset and proposed a novel model combined temporal feature based on RR interval and learned features to classify arrhythmia, the F_1_ score is 99.15% for super-ventricular ectopic beats and 95.2% for ventricular-ectopic beats. Khatibi et al. (Khatibi and Rabinezhadsadatmahaleh, 2019) proposed a novel feature engineering method based on deep learning and K-NNs showing a good performance to classify heartbeat. With the use of five k-fold cross-validation strategy, they achieved 99.99% average AUC, 99.30% recall. In their study, Hanbay (Hanbay, 2019) calculated six statistical features of ECG beat intervals and proposed a classification method based on eigenvalues and deep learning to evaluate ECG beats classes, which obtained an overall accuracy for N, S, V, and F (definition according to AAMI EC57 standard, as shown in Table 1) as 99.51% in classification. Wang et al. (Wang et al., 2020) attempted the use of four-channel of ECG as vector representation of learning input in their models, achieving the F_1_ score of 92.38%. Smigiel et al. (Smigiel et al., 2021) carried out three neural network architectures on PTB-XL Database (Kooman et al., 2020; Wagner et al., 2020; Hong et al., 2021; Merdjanovska and Rashkovska, 2022), and the proposed convolutional network with entropy features achieved the highest accuracy in every classification task, scoring 89.2%, 76.5%, and 69.8% accuracy for 2, 5, and 20 classes, respectively. Huang et al. (Huang et al., 2019) proposed a 2-D CNN to classify ECG arrhythmia. They used time-frequency spectrograms of five heartbeat types as input to the CNN network. Their model obtained 99% averaged accuracy, showing a high accuracy without manual preprocessing of ECG signals. Wang et al. (Wang et al., 2019) established the Global Recurrent Neural Network (GRNN) classification model, which was combined with automatic feature learning and optimization mechanism, obtaining 99.8% accuracy on MIT-BIH database.

**TABLE 1:** ECG heartbeat classes according to ANSI/AAMI EC57.

MIT-BIH	Heartbeat types
N	•Normal
	•Left/Right bundle branch block
	•Atrial escape
	•Nodal escape
S	•Atrial premature
	•Aberrant atrial premature
	•Nodal premature
	•Supraventricular premature
V	•Premature ventricular contraction
	•Ventricular escape
F	•Fusion of normal and ventricular
Q	•Paced
	•Fusion of normal and paced
	•Unclassifiable

All of studies mentioned above showed outstanding ECG arrhythmia classification performance, but none of them focused the issues introduced by imbalanced datasets. Furthermore, these excellent algorithms do not fully addressed issues of both robust real-time and effectiveness. It is common that ECG data in these datasets are imbalanced, with some common cardiac arrhythmias having overwhelming data samples as compared to those of rare cardiac arrhythmic types. Such imbalanced data samples affect the training of the AI/ML models, affecting the overall performance of the developed models for multiple categorical arrhythmia classifications. However, the performance of minority classes is poor due to lack of data on certain abnormal heart rhythms being less common in real life. It is still difficult to deal with an imbalanced dataset using deep learning for multiple type classification of cardiac arrhythmias, which is highly dependent on data quantity and quality. Moreover, high noise and complexity of ECG make large amounts of demand for computing resources, and the fewer parameters and more robust models are highly desired for.

One of the objectives of this study was to tackle the issue(s) arising from imbalanced datasets, which affecting model performances. To solve the limitation of an imbalanced ECG dataset, in this study we pay more attention to minor categories and suggest a weight-based loss function to reduce the influence of over-weighted categories in the ECG arrhythmia dataset. In addition, the presented deep learning model takes advantages of CNN and RNN, which consists of fewer parameters allowing for less computing demand. Possible effect of hyperparameters, optimization function and activation function on model performance were also analyzed. The proposed algorithm in this paper presents fewer parameters of architecture and relative high performance as compared to contemporary algorithms (Guo et al., 2019).

The contributions of this paper are listed as follows.

• Develop a new model architecture. A model consisting of three depth-wise separable convolutional neural networks (CNN) is constructed first, followed by bidirectional long short-term memory (Bi-LSTM), which effectively combines the speed of CNN and sequential sensitivity of recurrent neural network (RNN).
• Weight factor is embedded in the loss function of the training and validation dataset to eliminate the deviation of arrhythmia classification caused by the unbalanced types of the ECG heartbeat.
• Analyze the influence of weight-based loss and different hyperparameters on the model, including the activation function and optimization function on the performance of the algorithm.

The remainder of the paper is organized as follows. *Introduction* presents the motivation and literature review. The experimental setup includes dataset, preprocessing steps, hardware, software resources and methods are described in *Materials and methods*. *Results* part describes performance evaluation indicators, and experimental results of the proposed deep learning architecture. *Discussion* section discusses the compared performance with/without weigh-based loss, different activation functions, optimizer, and results proposed by other algorithms. *Limitations* part summarizes limitations of this work and possibilities for future research. Finally, a brief conclusion is shown in *Conclusion* section.

## Materials and methods

### Hardware and software

The deep learning framework adopts Keras (Chollet, 2018) with Tensorflow (Abadi et al., 2016) as the backend deep learning library. The constructed deep learning models are trained on the computer with one CPU running at 3.89GHz, an NVIDIA GeForce GTX 1660 GPU, and 8 Gb of memory.

### Dataset

In this study, MIT-BIH Arrhythmia Database (Goldberger et al., 2000) is used to develop ECG classification model. It is the most commonly used database, allowing cross comparison of experiment results. For MIT-BIH Arrhythmia Database, each record collects about 48 (Male 25: Female 22, Ages: 23–89 years) fully 30-min 2-lead ECG (electrodes positioned on the chest to obtain modified lead II and lead V_1_) with 360 Hz sampling rate and an expert annotated file.

Arrhythmia Database contains a variety of heartbeat types. In this study, according to ANSI/AAMI EC57 standard (I.A. American National Standards Institute, 1998), we classify ECG heartbeat into five groups based on annotation files. Table 1 provides a list of definition and specification of the five types of cardiac rhythms, and their corresponding labeling.

The dataset sample was preprocessed with the same steps as proposed by Kachuee et al. (Kachuee et al., 2018) before inputting them into the deep learning model. The specific steps are listed below:

(1) Resample the ECG signals as 125 Hz sampling frequency.
(2) Divide continuous ECG signals into 10 s window and normalize them in amplitude.
(3) Find the set of all local maximums based on the first derivative, and take 0.9 of normalized maximums as threshold for R peak candidates.
(4) The median of all R-R time intervals is taken as the nominal heartbeat period of this time window (*T*), and the length of each segment is determined as 1.2*T* for each R-peak, the rest is padded zeros to achieve the same length.

**FIGURE 1:**
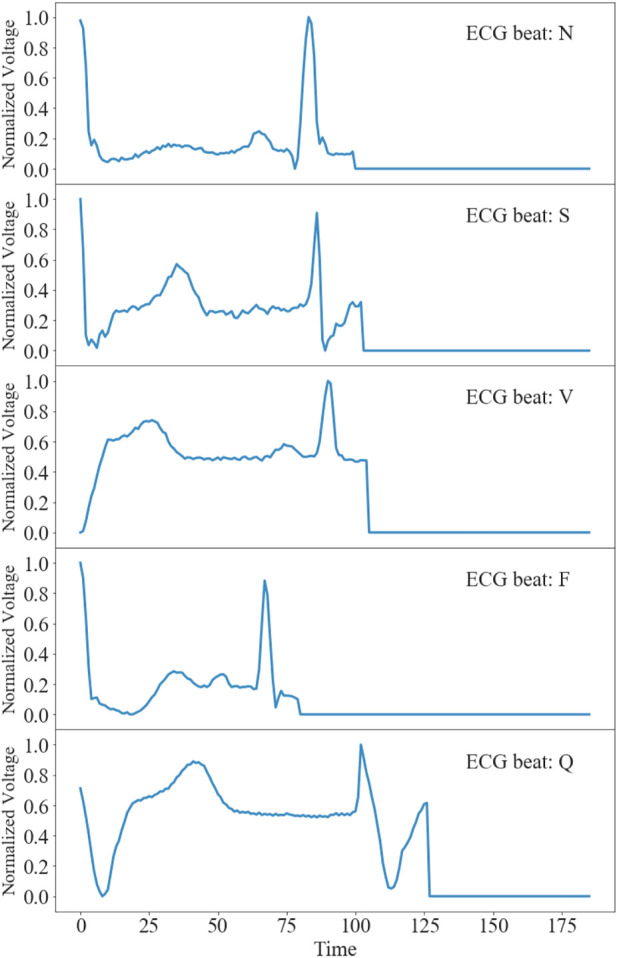
Time traces of representative ECGs for five types of heartbeats after preprocessing in the MIT-BIH Arrhythmia Database (Goldberger et al., 2000).

**FIGURE 2:**
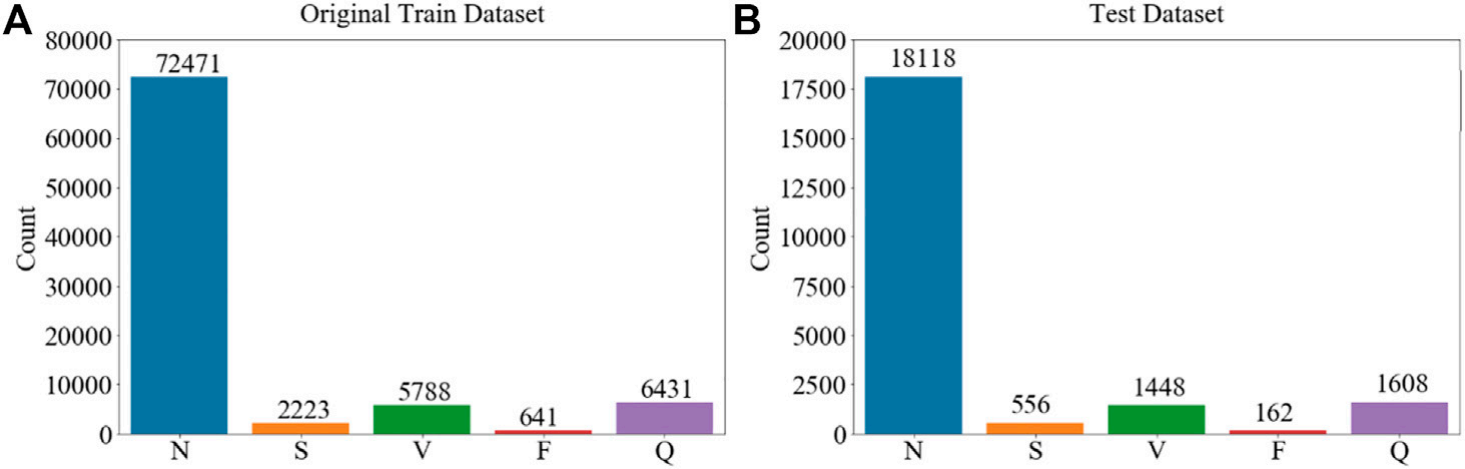
Sample population’s distribution of five types of ECG heartbeat in the MIT-BIH Arrhythmia Database (Goldberger et al., 2000) used in the study according to AAMI EC57. **(A)** Training dataset. **(B)** Test dataset. Imbalanced dataset for different types of cardiac rhythms is shown.

Figure 1 shows representative time traces of ECGs for five types of heartbeat randomly sampled from the training database after preprocessing. The training and test data samples after preprocessing have 87,554 and 12,892 sets of samples respectively. The population distributions of the five types of heartbeat for the training and test datasets are shown in Figure 2. The minor F heartbeat (641 in training dataset, 162 in test dataset) is less than one percent of the largest N heartbeat (72,471 in training dataset, 18,118 in test dataset), and the sum of all abnormal heartbeat classes is only about one-fifth of N heartbeat class. It is obvious that the MIT-BIH Arrhythmia dataset is unbalanced, with sample number for abnormal heartbeat being much less for rare abnormal heartbeats.

### Methods

**FIGURE 3:**
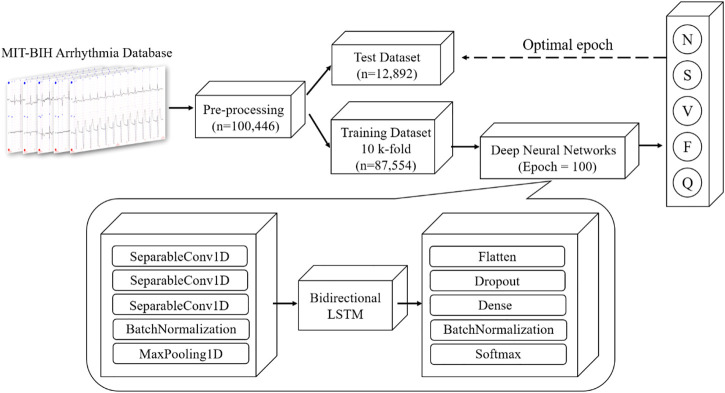
Overview architecture of proposed CNN + Bidirectional LSTM model for five types of heartbeat, N, S, V, F and Q (definition is provided in Table 1).

The schematic illustration of the classification model of this study is illustrated in Figure 3. Our proposed CNN + bidirectional LSTM model is composed of three steps. Firstly, five types of ECG heartbeat signals after preprocessing in the training dataset are firstly input into the one-dimensional CNN model consisting of three separable convolution layers. Secondly, apart from CNN, bidirectional LSTM is used to analyze sequential ECG, which is proven to be able to learn the sequential features from ECG contexts forward and backward (Andersen et al., 2019). Finally, the dense connection module gives the results of ECG heartbeat classification according to the learned features. This deep learning module is combined with the speed of CNN and the sequential sensitivity of RNN (Chollet, 2018), and high-level features can be extracted and contribute to achieve high classification accuracy.

To avoid the defect of data leakage in the process of model validation and reduce the generalization performance of the model, the training and validation of the model are carried out on the dataset as shown in Figure 2A, the trained model is tested only once on the test dataset as shown in Figure 2B. The popular 10 k-fold cross-validation technique is adopted in this study to overcome the overfitting issue and affirm the robustness (Chollet, 2021). The training dataset is divided into 10 subsets each of size *N*/10, the subset *i* is implemented as validation dataset, while the remaining nine subsets are used for training. The final performance score is computed as the average of 10 cross-validations.

As stated above, the sample number of ECG heartbeats of the five types of the heartbeat in the MIT-BIH Arrhythmia Database is extremely unbalanced (see Figure 2). This may cause the training of the model being heavily inclined towards the majority class through the back propagation of the loss function, resulting in biased and poor performance of the model for minority classes. To avoid the calculated loss function being mainly dominated by the large sample category of the unbalanced dataset, in this study we implemented a weight factor to scale the loss function of the training and validation datasets as shown in below equation, where *n_samples* represents the total number of the dataset used for training, *n_classes* represents five ECG heartbeat categories, and *np. bincount(y)* represents the specific number for each ECG heartbeat. In this way, the category weight factor of a small sample is high, while that of a large sample is low.

$$Weight=\frac{n\_samples}{n\_classes\times np.bincount\left(y\right)}$$

In implementation, we used an adaptive learning rate based on the computed value of loss function, i.e., reducing the learning rate to half of the previous value if the loss function does not decrease for five consecutive training epochs. The above strategy is employed to train the model for 100 epochs, during which the optimal epoch with a minimum loss function was identified. With the optimal epoch, the model was re-initialized and trained on the total training dataset, then this model was evaluated only once on the test dataset. It aims for preventing information leaks into the model due to multiple validation processes, which reduces the reliability of the model, even though the model is not directly trained on validation data.

The specific parameters of each layer are depicted in Table 2. The depthwise separable convolution includes depthwise convolution and pointwise convolution. Depthwise convolution performs separable convolution on each channel of input ECG signals to blend temporal features and pointwise convolution mixes the output channel by 1 × 1 convolution to blend channel features. This leads to fewer parameters and less computational cost, resulting in a smaller and faster neural network. Each of three separable CNN layers with ReLu activation function (Nair and Hinton, 2010) has 32, 64, and 128 kernels of size three respectively. The CNN can transform the long input sequence into a shorter sequence composed of much higher and more abstractive features. After convolution networks, a batch normalization layer is utilized to make the mean and variance change with time in training, and standardize the data adaptively. The batch normalization is helpful for model learning and the generalization of new data samples (Ioffe and Szegedy, 2015). After the batch normalization layer, a max-pooling layer with a parameter of 2 × 1 and strides two is applied to down-sample these features. This help to reduce the number of processed features and make a larger observation window of CNN, thus achieving the hierarchical structure of spatial filters. Then, the bidirectional LSTM layer with 128 units is utilized to solve the gradient disappearance problem. Bidirectional LSTM is employed to present the same information in different ways to model and improve the accuracy of this algorithm. Finally, the predicted model consists of a flattening layer, dropout layer, dense connection, batch normalization layer, and Softmax layer to predict the heartbeat class probability of the ECG. The first dense layer has 512 neurons and also uses the ReLu activation function. The 50% features are set to zero in the dropout layer to prevent overfitting (Gal, 2016).

**TABLE 2:** The structure of the constructed CNN + Bi-LSTM model.

No.	Layer name	(Kernel, Stride)	Output shape	Parameters
1	SeparableConv1D	(3,1)	(185, 32)	67
2	SeparableConv1D	(3,1)	(185, 64)	2,208
3	SeparableConv1D	(3,1)	(181, 128)	8,512
4	BatchNormalization	-	(181, 128)	512
5	Maxpooling1D	(2,2)	(90, 128)	0
6	Bidirectional LSTM	-	(256)	263,168
7	Flatten	-	(256)	0
8	Dropout	-	(256)	0
9	Dense	-	(512)	131,584
10	BatchNormalization	-	(512)	2,048
11	Dense	-	(5)	2,565

The model is compiled using the Adam optimizer (Kingma and Ba, 2015) and categorical cross-entropy loss function. The total parameters of the proposed model are 410,664, there are 409,384 trainable parameters and 1,280 non-trainable parameters introduced by the batch-normalization layer.

## Results

### Performance evaluate

To measure the overall performance of the proposed algorithm, the following performance metrics are suggested: Accuracy (Acc), Sensitivity (Sen), Specificity (Spe), Positive Prediction (PPV), and F_1_ score.

$$Acc=\frac{TP+TN}{TP+FP+FN+TN}$$

$$Sen=\frac{TP}{TP+FN}$$

$$Spe=\frac{TN}{TN+FP}$$

$$PPV=\frac{TP}{TP+FP}$$

$$F_{1}=\frac{2\times PPV\times Sen}{PPV+Sen}$$

Where *TP* is the number of true positives, *TN* is the number of true negatives, and *FP* and *FN* are the numbers of false positives and false negatives respectively.

From the above equation, accuracy indicates the percentage of the correct number predicted in the total dataset and can be used to judge the accuracy of the model. However, the MIH-BIH Arrhythmia dataset is imbalanced, accuracy cannot be used as a good indicator to measure the performance of the proposed algorithm. Therefore, another two indicators: Sensitivity (Sen), Specificity (Spe) and Positive Prediction (PPV) are used to measure the performance of the algorithm. Sensitivity (Sen), also known as recall rate, refers to the probability of being predicted to be a positive sample in a sample that is positive. Specificity (Spe) illustrates the proportion of negative cases identified to all negative cases. Positive Prediction Value (PPV) represents the probability of actually being positive out of all samples predicted to be positive. To find the balance between Sen and PPV, the F_1_ score is developed, both Sen and PPV are considered to achieve the maximum at the same time.

**FIGURE 4:**
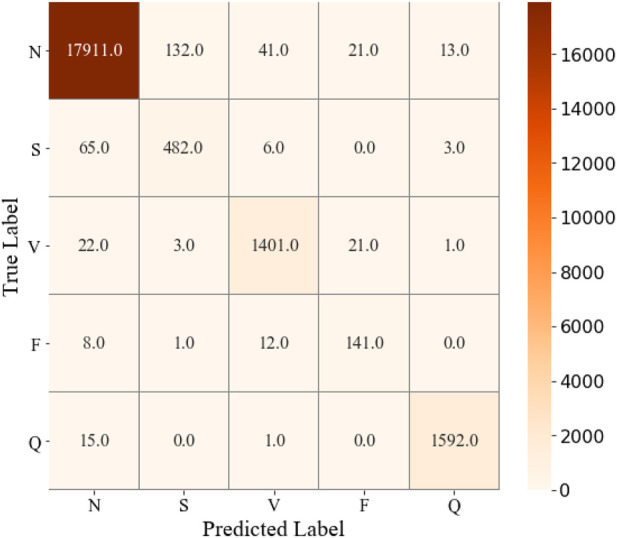
Confusion matrix of ECG classification results on the test dataset by performing CNN + Bi-LSTM model.

**FIGURE 5:**
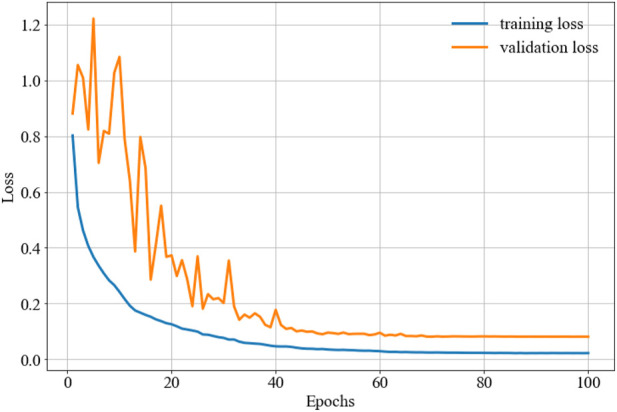
The training and validation loss.

**FIGURE 6:**
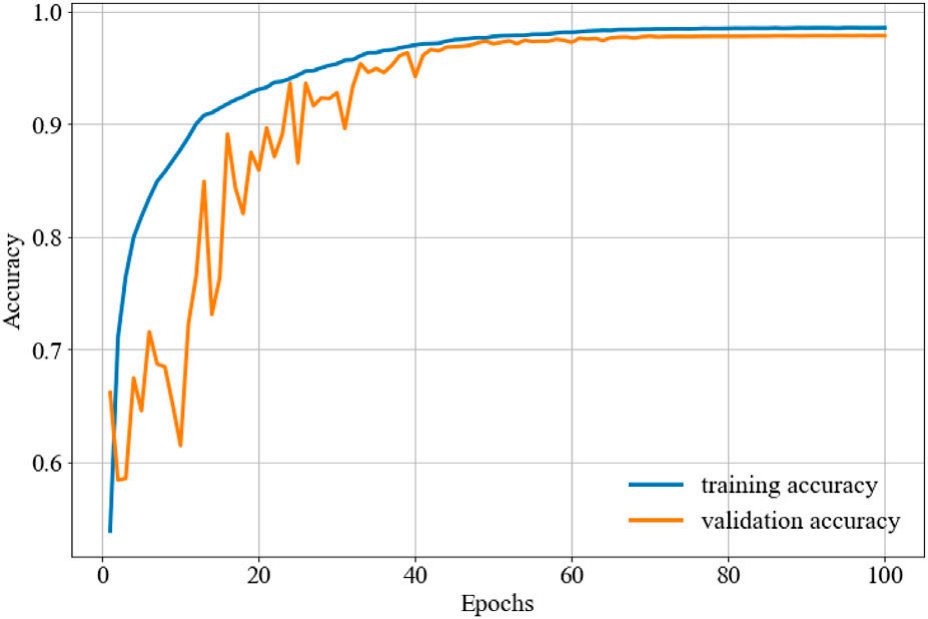
The training and validation accuracy.

The parameter configuration in this study is the ReLu activation function (Nair and Hinton, 2010), and the Adam optimization function (Kingma and Ba, 2015). Furthermore, the 128 batch size yields better performance than other schemes. The confusion matrix can be seen in Figure 4, the training and validation loss is shown in Figure 5, and the accuracy of the training and validation dataset in Figure 6. Table 3 shows the Acc, Sen, Spe, PPV, and F_1_ score for each heartbeat class, and the averaged results are shown in bold. It is clear that Sen, PPV, and F_1_ score should be given more attention for imbalanced dataset.

**TABLE 3:** Performance results per heartbeat class, and averages are given in bold.

MIT-BIH	Acc (%)	Spe (%)	Sen (%)	PPV (%)	F_1_ (%)
N	98.55	97.09	98.86	99.39	99.12
S	99.04	99.36	86.69	77.99	82.11
V	99.51	99.71	96.75	95.89	96.32
F	99.71	99.81	87.04	77.05	81.74
Q	99.85	99.92	99.00	98.94	98.97
Average	**99.33**	**99.18**	**93.67**	**89.85**	**91.65**

## Discussion

In this section, we ablated the influences of the uses of the weight-based loss, k-fold, activation function and optimization function in the model performance in detail. Additionally, we also compared experimental results of the proposed results with other studies, of which are shown in Table 8, where the best performances for each index are shown in boldface.

### Performance evaluation under model with weight-based loss

The impact of proposed weight-based loss was measured in our approach as shown in Table 4, in which the best results were shown in boldface. It was clear that the method without weight-based loss dropped off significantly in accuracy (99.33%–92.26%), specificity (99.18%–98.75%), sensitivity (93.67%–93.51%), positive prediction value (89.85%–74.24%) and F_1_ score (91.65%–80.57%). Especially, the biggest boost in F class was resulted from the adoptive weight-based loss in the imbalanced dataset.

**TABLE 4:** Classification performance results with and without weight-based loss. The best performances are given in bold.

Heartbeat types	With Weight-based Loss					Without Weight-based Loss				
	Acc (%)	Spe (%)	Sen (%)	PPV (%)	F_1_ (%)	Acc (%)	Spe (%)	Sen (%)	PPV (%)	F_1_ (%)
N	**98.55**	97.09	**98.86**	99.39	**99.12**	95.91	**97.85**	95.50	**99.53**	97.48
S	**99.04**	**99.36**	86.69	**77.99**	**82.11**	98.31	98.59	**87.59**	61.80	72.47
V	**99.51**	**99.71**	96.75	**95.89**	**96.32**	99.33	99.47	**97.31**	92.88	95.04
F	**99.71**	**99.81**	87.04	**77.05**	**81.74**	98.02	98.09	**87.65**	25.54	39.55
Q	**99.85**	**99.92**	99.00	**98.94**	**98.97**	99.75	99.77	**99.50**	97.14	98.31
Average	**99.33**	**99.18**	**93.67**	**89.85**	**91.65**	98.26	98.75	93.51	74.24	80.57

### Performance evaluation of the model with 10 k-fold

**FIGURE 7:**
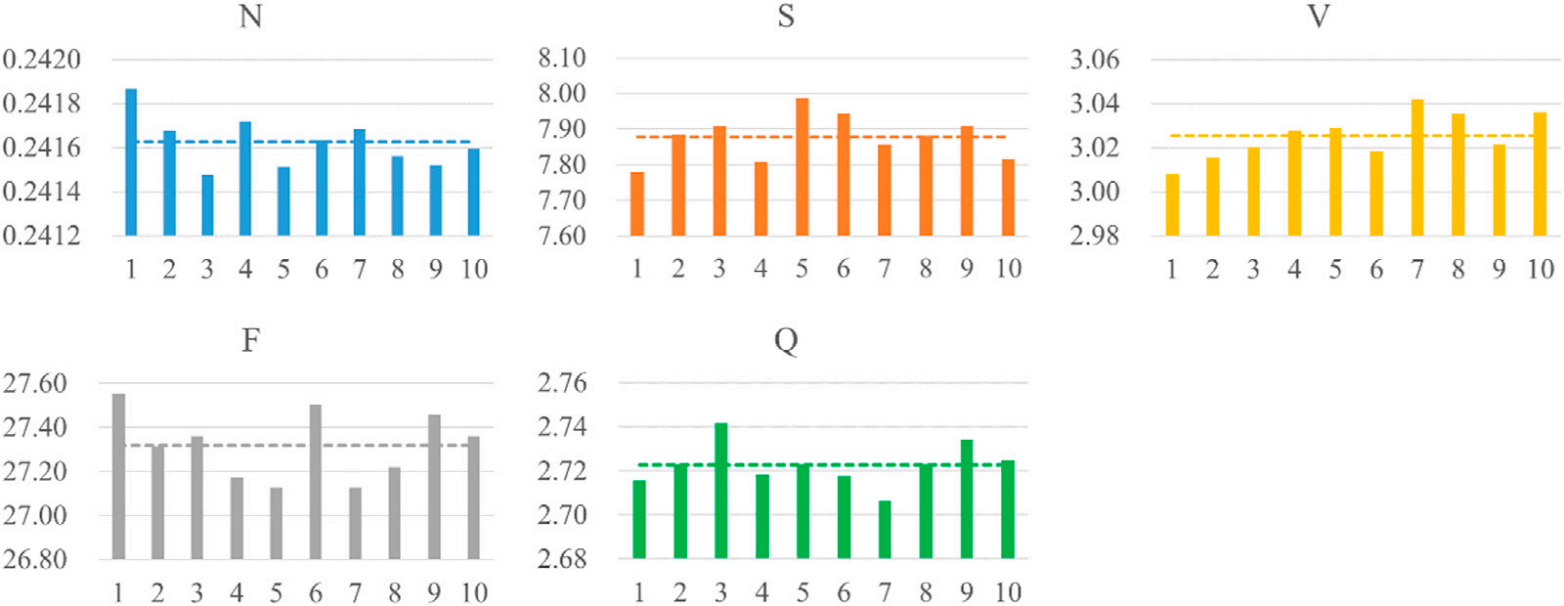
Distribution of weight-based loss factor for five heartbeats per fold. The dashed line shows the averaged weight-based loss factor.

The popular 10 k-fold cross-validation was employed in our study. As shown the compared results in Table 5, the 10 k-fold measurements are slightly better than not using cross-validation method. We know that different division of training and validation dataset leads to a large fluctuation of validation scores, which in turn causes variance and unreliability on results. Therefore, the common 10 k-fold cross-validation was applied. Figure 7 depicts the distribution of weight factors with different folds and the mean weight-based loss factor (dashed line) for each heartbeat showing, N class contributed least in the total loss since the huge counts. Conversely, minor heartbeat categories contributed more in the total weight factor of loss.

**TABLE 5:** Comparison of experimental results with and without 10 k-fold. The best performances are given in bold.

Heartbeat types	10 k-fold					Without k-fold				
	Acc (%)	Spe (%)	Sen (%)	PPV (%)	F_1_ (%)	Acc (%)	Spe (%)	Sen (%)	PPV (%)	F_1_ (%)
N	**98.55**	97.09	**98.86**	99.39	**99.12**	98.53	**97.35**	98.77	**99.44**	99.11
S	99.04	**99.36**	86.69	**77.99**	82.11	**99.05**	**99.36**	**87.23**	77.97	**82.34**
V	**99.51**	**99.71**	96.75	**95.89**	96.32	**99.51**	99.69	**97.03**	95.64	**96.33**
F	**99.71**	**99.81**	87.04	**77.05**	**81.74**	99.63	99.72	**88.27**	70.10	78.14
Q	99.85	99.92	**99.00**	98.94	98.97	**99.88**	**99.95**	98.94	**99.38**	**99.16**
Average	**99.33**	99.18	93.67	**89.85**	**91.65**	99.32	**99.21**	**94.05**	88.51	91.02

### Different activation function

To evaluate the performance of different activation functions on the proposed algorithm, we compared performance of the designed model using ReLu (Nair and Hinton, 2010) and Elu activation function (Clevert et al., 2016). The best performances for each heartbeat type are shown in boldface in Table 6. Overall, the ReLu has better experimental results in this study although Elu is better than ReLu in theory (Clevert et al., 2016).

**TABLE 6:** Experimental results between ReLu and Elu activation function. The best performances are given in bold.

Heartbeat types	Relu					Elu				
	Acc (%)	Spe (%)	Sen (%)	PPV (%)	F_1_ (%)	Acc (%)	Spe (%)	Sen (%)	PPV (%)	F_1_ (%)
N	**98.55**	97.09	**98.86**	99.39	**99.12**	98.47	**97.14**	98.75	**99.40**	99.08
S	99.04	99.36	**86.69**	77.99	82.11	**99.08**	**99.42**	85.97	**79.40**	**82.56**
V	**99.51**	**99.71**	**96.75**	**95.89**	**96.32**	99.42	99.63	96.48	94.84	95.65
F	**99.71**	**99.81**	87.04	**77.05**	**81.74**	99.66	99.72	**90.74**	71.01	79.67
Q	99.85	99.92	99.00	98.94	98.97	**98.87**	**99.93**	**99.19**	**99.07**	**99.13**
Average	**99.33**	**99.18**	93.67	**89.85**	**91.65**	99.10	99.17	**94.23**	88.74	91.22

### Different optimizer

In deep learning, the optimizer affects the speed and mode of convergence of the algorithm. Adam optimizer is the combination of SGDM (SDG with Momentum) and RMSProp (Kingma and Ba, 2015). As shown in Table 7, the best performances are shown in boldface, the F_1_ score experimental results with Adam are better than RMSProp. It turned out that the Adam optimizer resulted in a significant enhancement in Sen (88.79%–93.67%), PPV (65.13%–89.85%) and F_1_ score (81.88%–91.65). In particular, a 25% improvement in PPV was observed by using Adam.

**TABLE 7:** Experimental results between Adam and RMSProp activation function. The best performances are given in bold.

Heartbeat types	Adam					RMSProp				
	Acc (%)	Spe (%)	Sen (%)	PPV (%)	F_1_ (%)	Acc (%)	Spe (%)	Sen (%)	PPV (%)	F_1_ (%)
N	**98.55**	**97.09**	**98.86**	**99.39**	**99.12**	90.37	96.16	89.17	99.11	93.88
S	**99.04**	**99.36**	**86.69**	**77.99**	**82.11**	95.07	95.43	80.94	31.60	45.45
V	**99.51**	**99.71**	96.75	**95.89**	**96.32**	96.09	96.00	**97.38**	63.29	76.71
F	**99.71**	**99.81**	**87.04**	**77.05**	**81.74**	98.62	98.74	82.10	32.76	46.82
Q	**99.85**	**99.92**	**99.00**	**98.94**	**98.97**	99.51	99.92	94.34	98.89	96.56
Average	**99.33**	**99.18**	**93.67**	**89.85**	**91.65**	95.93	97.25	88.79	65.13	81.88

### Comparison with other algorithms

Table 8 summarizes the comparison of experimental results from the proposed algorithm on five heartbeat types and other published researches on the MIT-BIH database. The best performances for each evaluation index are shown in bold font. It is worth noting that the algorithm proposed by Zahid et al. (Zahid et al., 2022) showed the best specificity 99.83% for N heartbeat type, and Sellami et al. (Sellami and Hwang, 2019) presented the higher accuracy 99.99% and specificity 89.54% for S heartbeat type. However, the specificity and accuracy are not the reasonable measure indexes in imbalanced dataset as shown in Table 8. The F_1_ score should be considered as the most noteworthy performance metric in the paper. Our proposed algorithm gave the best performance in five heartbeat types for F_1_ score in Table 8. Despite the results in our presentation so far were not the best performance because of we aimed at the lightweight model parameters such as including noise in raw signals, and tested them only once on test dataset to avoid information leakage and thus reduced the credibility of the model. However, it is a good attempt to balance efficiency and robustness in ECG heartbeat classification.

**TABLE 8:** Compared experimental results of our approach and other algorithms on MIT-BIH dataset. The best performance is shown in bold.

Algorithm	N			S			V			F			Q		
	Acc (%)	Spe (%)	F_1_ (%)	Acc (%)	Spe (%)	F_1_ (%)	Acc (%)	Spe (%)	F_1_ (%)	Acc (%)	Spe (%)	F_1_ (%)	Acc (%)	Spe (%)	F_1_ (%)
Proposed	**98.55**	97.09	**99.12**	**99.04**	**99.36**	**82.11**	**99.51**	**99.71**	**96.32**	**99.71**	**99.81**	**81.74**	99.85	99.92	**98.97**
Mathews et al. (2018)	---	---	---	93.78	93.32	48.72	96.63	97.83	73.22	---	---	---	---	---	---
Kiranyaz et al. (2016)	---	---	---	97.6	99.2	61.86	99.0	98.9	92.22	---	---	---	---	---	---
Niu et al. (2020)	---	---	98.10	---	---	76.60	---	---	89.70	---	---	---	---	---	---
Guo et al. (2019))	---	---	---	93.61	96.40	61.94	93.71	94.77	89.75	---	---	---	---	---	---
Zahid et al. (2022)	---	**99.83**	99.07	---	**99.36**	83.44	---	89.76	94.29	---	---	---	---	---	---
Wang et al. (2021)	---	---	98.79	98.74	---	81.37	99.27	---	94.43	---	---	0.46	---	---	---
Li et al. (2022)	---	80.8	93.93	---	98.83	45.90	---	94.92	83.89	---	---	---	---	---	---
Sellami and Hwang, (2019)	88.82	91.30	93.37	92.41	92.80	44.40	97.18	97.54	80.88	92.28	98.52	38.27	**99.99**	**100**	22.23

## Limitations

ECG is a reflection of potential change of cardiac tissue during the propagation of cardiac excitations through measuring body surface potential in different parts of the body. Changes in morphology and characteristics of ECG can be used to detect abnormal rhythm of the heartbeat (e.g., cardiac arrhythmia). The computer-aided ECG diagnosis system may add a great value to interpret complicated ECG signals. The application of artificial intelligence for ECG interpretation is highly concerned, deep learning with the advantage of gaining high-level features can contribute to high ECG classification accuracy. The proposed deep learning algorithm combined with separable CNN and bidirectional LSTM offers an automatic classification of ECG heartbeat. The limitations and opportunities of the present study for future work as listed below:

(i) To reduce the computational burden and improve efficiency, we implemented a simple segment splitting operation of ECG signals without denoising in our preprocessing stage. However, ECG signals contain various noises in reality, such as baseline wander and power line interference (Sharma and Pachori, 2018; Liu et al., 2021). Deep learning relies heavily on data quality, high noises and complexity of ECG signals reduce the performance of the proposed deep learning model due to irrelevant noise information being learned by deep learning. Having much cleaner and denoised input signals can improve deep learning by discovering more abstractive features. Subsequent research should consider applying denoised methods including Fourier transform, cut-off frequency, and so on.
(ii) Another problem is the data imbalance. Unbalanced signal types are common in all ECG datasets due to abnormal heartbeats that are hard to be collected in practice. Less abnormal samples indicate worse sensitivity and positive predication than the large number ECG heartbeats. In this study, a weight factor was used to scale the loss function to reduce the effect of the unbalanced dataset. Some previous studies come up with innovative ideas, including a special focus on minor classes (Sayantan et al., 2018; Rahhal et al., 2016; Tan et al., 2018) and the special architecture of the model (Sayantan et al., 2018; Hammad et al., 2021b; Jiang et al., 2019). In addition, Generative Adversarial Networks (GAN) (Goodfellow et al., 2014) were developed to augment minor ECG types, and also help to denoise ECG signals. Hence, these methods can be considered as possible attempts to solve this issue in the future.
(iii) In this work, MIT-BIT Arrhythmia Database is used to study classification. However, it was collected more than 40 years ago, the amount of abnormal heart rhythms is insufficient, and the different durations and leads make it unfair to compare with research on other databases. This limits a certain the generalization and robustness of the architectural model. Testing the constructed algorithm on other ECG databases can serve as an opportunity for future research.
(iv) The 10 k-fold method is adopted to eliminate bias and improve the robust in our approach, but it leads to expensive computation and further research is needed. In addition, due to the fewer parameters (410,664 parameters of the proposed model) and simple preprocessing steps in our method, the proposed algorithm may have certain advantages in clinical applications. However, the robust and efficiency of our research have not tested it clinically, which is what we need to continue to study in the future.

Although the proposed model does not perform the best among all AI methods, it is a new attempt to explore the high efficiency and robustness of algorithms in ECG heartbeat classification.

## Conclusion

In this paper, CNN-bidirectional LSTM model has been developed for the automated heartbeat classification of ECG signals. In addition, the ReLu activation function and Adam optimization function are implemented in this model. We pay more attention to minorities in the dataset by using a weight factor to scale the loss function to overcome data imbalance issue. Moreover, to prevent information leakage of data that leads to mistaken high performance, 10 k-fold cross-validations on the training dataset were conducted for training and validation, and the test dataset was used only once on the optimized model to prevent information leakage. The designed algorithm is shown to be useful in improving the F_1_ score of minor types of ECG heartbeat, resulting in an average of 99.33% accuracy, 99.18% specificity, 93.67% sensitivity, 89.85% positive prediction, and 91.65% F_1_ score. The proposed model shows more sensitivity than some other studies in ECG classification. In conclusion, we have developed a deep learning algorithm by a new attempt to overcome the dataset imbalance of ECG, resulting in a model with high efficiency and fewer parameters. It may serve as a potential tool for aiding ECG detection and classification.

## Data Availability

The original contributions presented in the study are included in the article/supplementary material, further inquiries can be directed to the corresponding author.
